# Linking Microbial Community Structure to Trait Distributions and Functions Using Salinity as an Environmental Filter

**DOI:** 10.1128/mBio.01607-19

**Published:** 2019-07-23

**Authors:** Kristin M. Rath, Arpita Maheshwari, Johannes Rousk

**Affiliations:** a Section of Microbial Ecology – MEMEG, Department of Biology, Lund University, Lund, Sweden; b Centre for Environmental and Climate Research (CEC), Lund University, Lund, Sweden; CEH-Oxford

**Keywords:** community ecology, biogeochemistry, predictive ecology, soil biology, soil carbon, soil microbiology, structure and function, traits

## Abstract

The structure and function of microbial communities vary along environmental gradients; however, interlinking the two has been challenging. In this study, salinity was used as an environmental filter to study how it could shape trait distributions, community structures, and the resulting functions of soil microbes. The environmental filter was applied by salinizing nonsaline soil (0 to 22 mg NaCl g^−1^). Our targeted community trait distribution (salt tolerance) was determined with dose-response relationships between bacterial growth and salinity. The bacterial community structure responses were resolved with Illumina 16S rRNA gene amplicon sequencing, and the microbial functions determined were respiration and bacterial and fungal growth. Salt exposure quickly resulted in filtered trait distributions, and stronger filters resulted in larger shifts. The filtered trait distributions correlated well with community composition differences, suggesting that trait distribution shifts were driven at least partly by species turnover. While salt exposure decreased respiration, microbial growth responses appeared to be characterized by competitive interactions. Fungal growth was highest when bacterial growth was inhibited by the highest salinity, and it was lowest when the bacterial growth rate peaked at intermediate salt levels. These findings corroborated a higher potential for fungal salt tolerance than bacterial salt tolerance for communities derived from a nonsaline soil. In conclusion, by using salt as an environmental filter, we could interlink the targeted trait distribution with both the community structure and resulting functions of soil microbes.

## Abstract (precis)

Understanding the role of ecological communities in maintaining multiple ecosystem processes is a central challenge in ecology. Soil microbial communities perform vital ecosystem functions, such as the decomposition of organic matter to provide plant nutrition. However, despite the functional importance of soil microorganisms, attribution of ecosystem function to particular constituents of the microbial community has been impeded by a lack of information linking microbial processes to community composition and structure. Here, we apply a conceptual framework to determine how microbial communities influence ecosystem processes, by applying a “top-down” trait-based approach. By determining the dependence of microbial processes on environmental factors (e.g., the tolerance to salinity), we can define the aggregate response trait distribution of the community, which then can be linked to the community structure and the resulting function performed by the microbial community.

## INTRODUCTION

Large land areas of the globe are affected by high salt concentrations in soil (1). Through land-use change and mismanagement of irrigation in agriculture, the predominance of salt-affected soils is increasing. Salts accumulate in the topsoil, as groundwater and water used for irrigation evaporate (2, 3). Soil microorganisms are negatively affected by high salt concentrations, which is reflected in, e.g., decreased microbial functions such as respiration and growth after salt exposure (4–6). However, microorganisms can counteract some of the negative effects of salinity through physiological adaptations. Organisms can adapt their physiology through the synthesis of osmolytes (7) and changes in the composition of cell membranes (8, 9). In addition to physiological responses of the resident community, selection for more salt-tolerant species can lead to a shift in the taxonomic composition (10). The changes in both community composition together with physiological adaptations manifest as an increased community salt tolerance in response to salt exposure (4). As community salt tolerance increases, microbial process rates that were inhibited in response to acute salt exposure can, at least partially, recover. All this makes soil salinity an interesting and reversible environmental filter that can be used in controlled experiments to advance microbial ecology.

Fungi and bacteria are reported to be differently affected by salt exposure (6, 11). Generally, fungi are considered to be more resistant to short-term exposure to salinity (6). However, it is unclear whether an increased resistance to short-term exposure would indeed translate to a shift toward a system that is dominated more by fungi, as both increasing fungal dominance (11, 12) and increasing bacterial dominance (13, 14) in response to high soil salinity have been reported. While bacteria and fungi fulfill similar roles as decomposers of organic matter, they differ in the range of substrates they can decompose (15). Fungal and bacterial biomass also differ in their chemical composition (16) and nutrient content (17, 18). Thus, shifts in the relative contributions of fungi and bacteria to decomposition in response to salinity could have implications for carbon and nutrient dynamics in soil (18, 19).

A central challenge in ecology is interlinking the structure of biological communities with ecosystem functions. Recent progress on predicting ecosystem functions from plant community information has been generated from trait-based frameworks, where community responses to changing environmental conditions along with the ecosystem functional consequences of these responses have been mapped out by drawing on and using response and effect traits (e.g., reference 20). More recently, these ideas have even been placed in a phylogenetic framework for animals and plants (21). These ideas have also begun to be incorporated into microbial ecology, where the traits of microbial taxa have been used to estimate functions (e.g., 22). However, within microbial ecology, trait-based approaches are particularly challenging to apply in practice. Part of the challenge is that the vast majority of the community members are difficult or impossible to cultivate, and progress is slow (23, 24), making it difficult to estimate traits for most microbial taxa. In addition, microbial taxa do not function in isolation. Rather, they operate in multitrophic consortia forming complex food webs. Thus, even if complete lists of taxa along with trait information from pure culture isolates were available from an environment, it is doubtful whether this would provide sufficient information to predict any ecosystem functions, which are also measured on an aggregate community level without any obvious way to assign partial contributions to the taxa resolved. In this study, we applied a conceptual framework based on C. T. Webb et al. (25) to achieve an improved ability to infer ecosystem functions from microbial structural information by interlinking them with functional trait distributions. Rather than resolving traits for individual microbial taxa, we intentionally set out to characterize community-aggregated traits (*sensu* [26]; henceforth “community trait distributions”) and to link this information to the community structure and resulting functions (decomposition and microbial growth rates), thus targeting structure, trait distributions, and functions on the same level of organization. We set out to test how a selected community trait distribution (salt tolerance) was shaped by the application of an environmental filter (soil salinity) and how these responses were linked to the microbial community structure (species composition) and functions (respiration and microbial growth). In addition, we monitored the time scale at which trait distributions could respond to the application of salt as an environmental filter and how the link between community trait distributions and the environment influenced microbial functions over time. To ensure that the microbial community composition resolved by the DNA composition would reflect the community traits and resulting functions, we linked the final microbial community composition and trait distributions to the cumulative functions (growth and respiration) resulting during the colonization of plant litter-amended soil samples during 40 days. Our hypotheses were (i) that the targeted community trait distribution (salt tolerance) would be filtered by the environmental gradient and eventually align with its environment, but (ii) that the adjustment would be gradual and thus take longer to manifest when the environmental filter was stronger (i.e., at higher salinities). We also hypothesized (iii) that responses in community trait distributions (salt tolerance) would be driven by species turnover, and therefore would correlate well to community compositional differences at the end of the experiment. We further hypothesized (iv) that the microbial functions respiration and growth would decrease with increasing strength of the induced environmental filter (i.e., higher salinities) immediately after exposure, but that the functions would subsequently (within a few weeks) partially recover due to the gradual alignment of functional trait distributions (increased salt tolerance) to the new environmental conditions, driven by structural changes in the community. Our final hypothesis (v) was that bacteria would perform worse at higher salinities than fungi and that therefore bacterial growth would decrease more strongly with increasing salinity than would fungal growth.

## RESULTS

### Responses of bacterial salt tolerance to changes in soil salinity.

**FIG 1:**
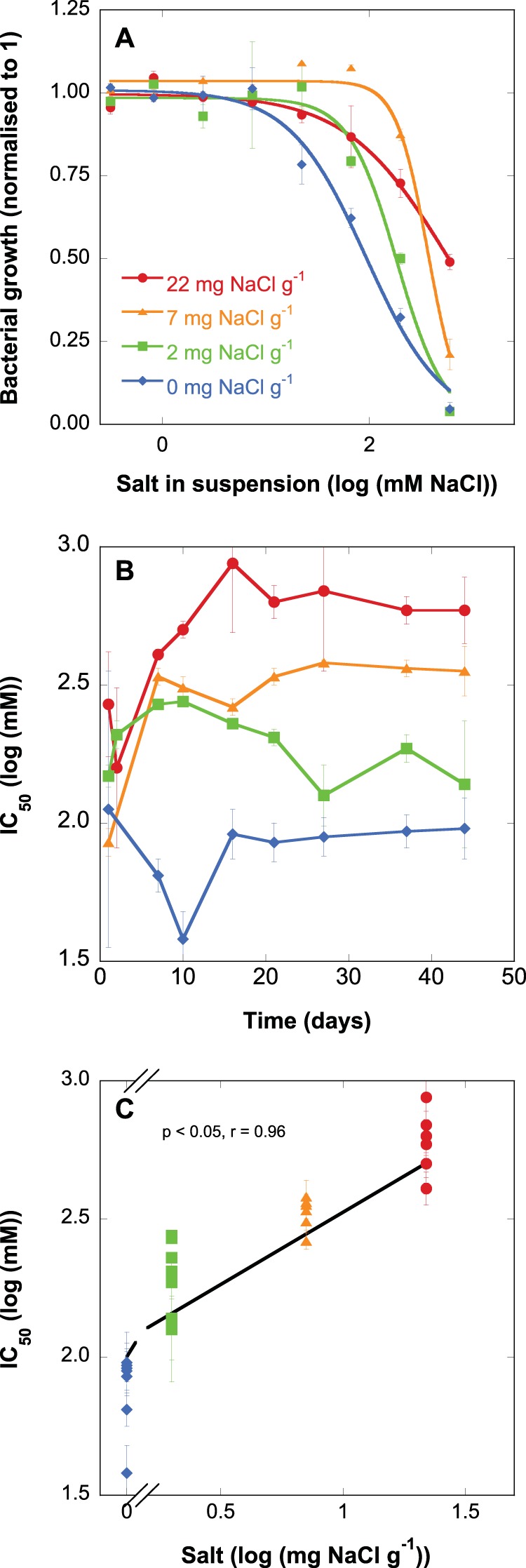
(A) Dose-response curves between salt concentration and bacterial growth at day 40 after salt addition. Bacterial growth was normalized to the maximum rate for each treatment. (B) Bacterial salt tolerance indexed as IC_50_ for bacterial growth in four different salt treatments (0, 2, 7, and 22 mg NaCl g^−1^) over time. (C) Bacterial salt tolerance indexed as IC_50_ for bacterial growth from day 10 against different salinity treatments. Values are means ± 1 standard error (SE) (error bars).

Bacterial tolerance to salt, indexed as the salt concentration that inhibited bacterial growth by 50% (IC_50_), started with similar values—corresponding to 100 to 200 mM NaCl in suspension in all levels of soil salinity—on day 0 (Fig. 1B). However, there was high variability in the measurements of bacterial salt tolerance during the first 3 days of the experiment. From ca. day 10 onwards, IC_50_ values were clearly lower in the 0 and 2 mg NaCl g^−1^ treatments than in the 7 and 22 mg NaCl g^−1^ treatments. The IC_50_ in the 0 mg NaCl g^−1^ treatment fluctuated around a level corresponding to 100 mM NaCl and in the 2 mg NaCl g^−1^ treatment around a level of 200 mM NaCl. The IC_50_ in the 7 mg NaCl g^−1^ treatment reached a level corresponding to ca. 300 mM NaCl on day 10 and remained constant after that. In the 22 mg NaCl g^−1^ treatment, the IC_50_ increased until a level of ca. 650 mM NaCl, which was reached only by day 20. Overall, bacterial salt tolerance increased with increasing salinity of the treatment (Fig. 1C).

### Responses of the bacterial community composition to changes in soil salinity.

**FIG 2:**
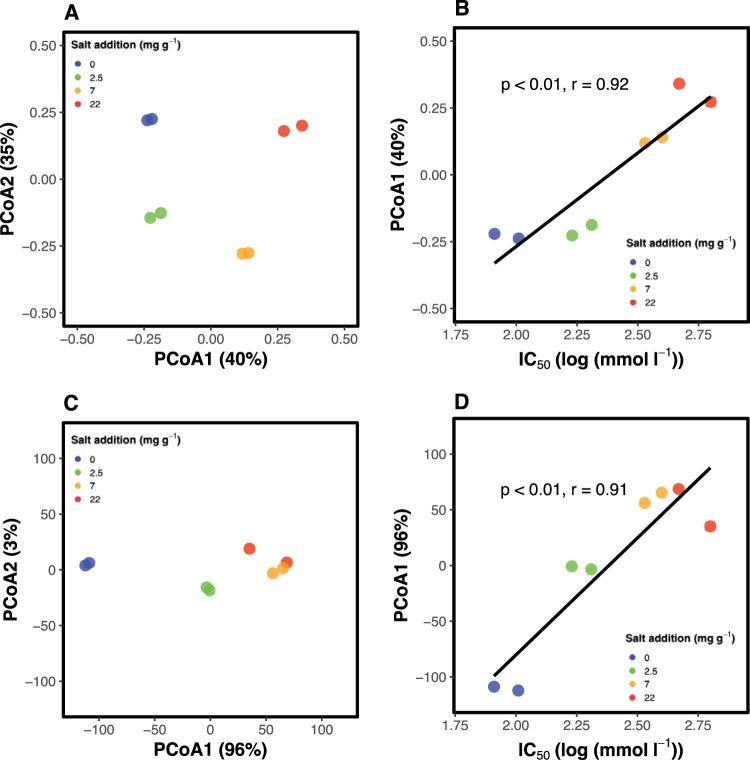
(A) Principal coordinate analysis (PCoA) of bacterial community composition based on Bray-Curtis dissimilarities of 16S rRNA amplicon sequencing data in four different salt treatments (0, 2, 7, and 22 mg NaCl g^−1^). (B) The first ordination axis of the Bray-Curtis dissimilarity-based PCoA against community salt tolerance indicated by the IC_50_. (C) Principal coordinate analysis (PCoA) of bacterial community composition based on Earth Mover Band Awareness Distances (EMBAD) of 16S rRNA amplicon sequencing data in four different salt treatments (0, 2, 7, and 22 mg NaCl g^−1^). (D) The first ordination axis of the EMBAD-based PCoA against community salt tolerance indicated by the IC_50_.

At the end of the experiment, salt exposure had resulted in pronounced differences in community composition between treatments (Fig. 2). In a principal coordinate analysis based on Bray-Curtis dissimilarities (Fig. 2), the four different salt treatments resulted in distinct communities, with similar community composition in duplicates of the same treatment. The first principal coordinate (PCo1) could be related to community salt tolerance (Fig. 2). An arch effect was visible in the ordination based on Bray-Curtis dissimilarities. In the alternative ordination based on EMBAD, the arch effect disappeared, and the first ordination axis accounted for 96% of the variation. In a Mantel test, bacterial community composition and community salt tolerance were significantly correlated with each other. This correlation was stronger still for EMBAD (ρ = 0.83, *P* < 0.01) than for Bray-Curtis dissimilarities of community composition (ρ = 0.61, *P* < 0.01). The taxa whose abundance was correlated with increasing community salt tolerance (Spearman’s ρ > 0.5) consisted primarily of *Firmicutes* (see Table S1 in the supplemental material). Out of the 17 operational taxonomic units (OTUs) correlated with community salt tolerance that had a maximum abundance of >0.5%, 9 belonged to the phylum *Firmicutes*, while the rest included members of the *Gammaproteobacteria*, *Actinobacteria*, *Bacteroidetes*, and *Verrucomicrobia*.

10.1128/mBio.01607-19.1 TABLE S1 Table of abundant OTUs (>0.5% of reads) that were positively correlated (Spearman’s rank correlation coefficient ρ > 0.5) with bacterial community salt tolerance. Download Table S1, DOCX file, 0.02 MB. Copyright © 2019 Rath et al. 2019 Rath et al. This content is distributed under the terms of the Creative Commons Attribution 4.0 International license.

### Responses of the microbial functions respiration and growth to changes in soil salinity.

**FIG 3:**
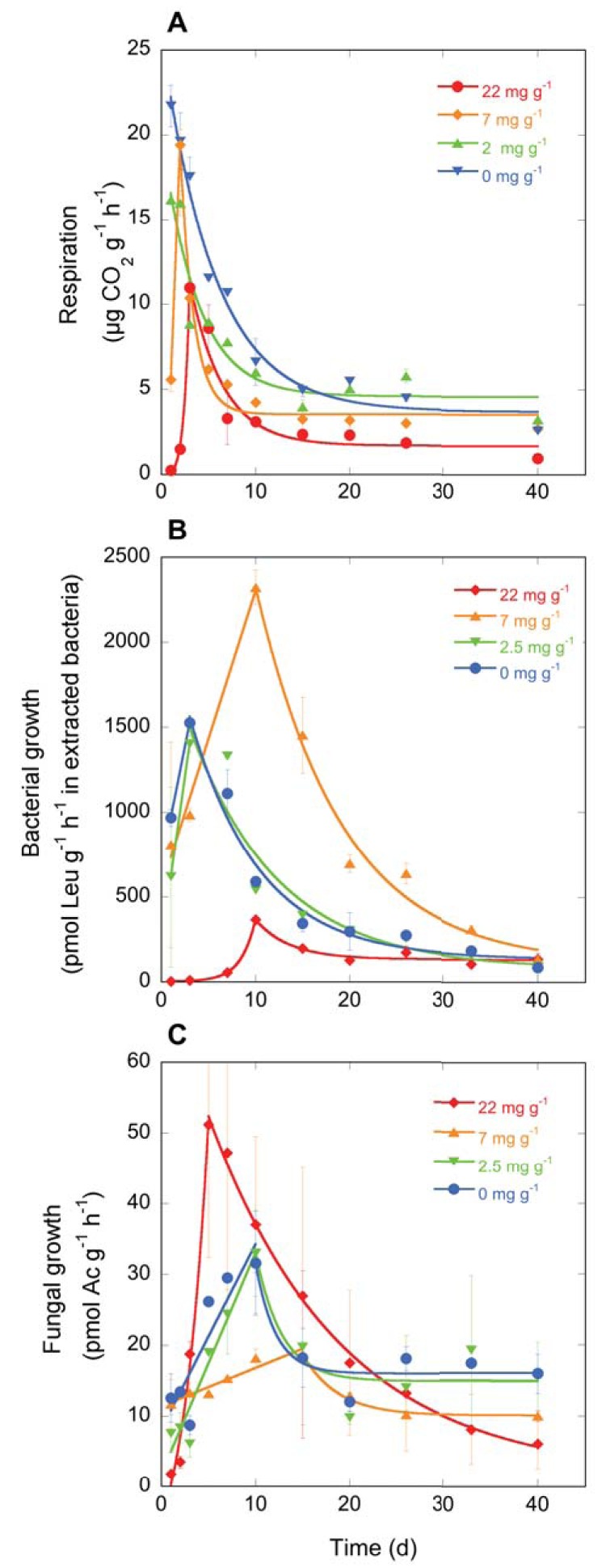
Respiration (A), bacterial growth (B) and fungal growth (C) rates in microcosms of four different salt treatments (0, 2, 7, and 22 mg NaCl g^−1^) with time after salt addition (in days). Values are means ± 1 standard error. Ac, acetate.

**FIG 4:**
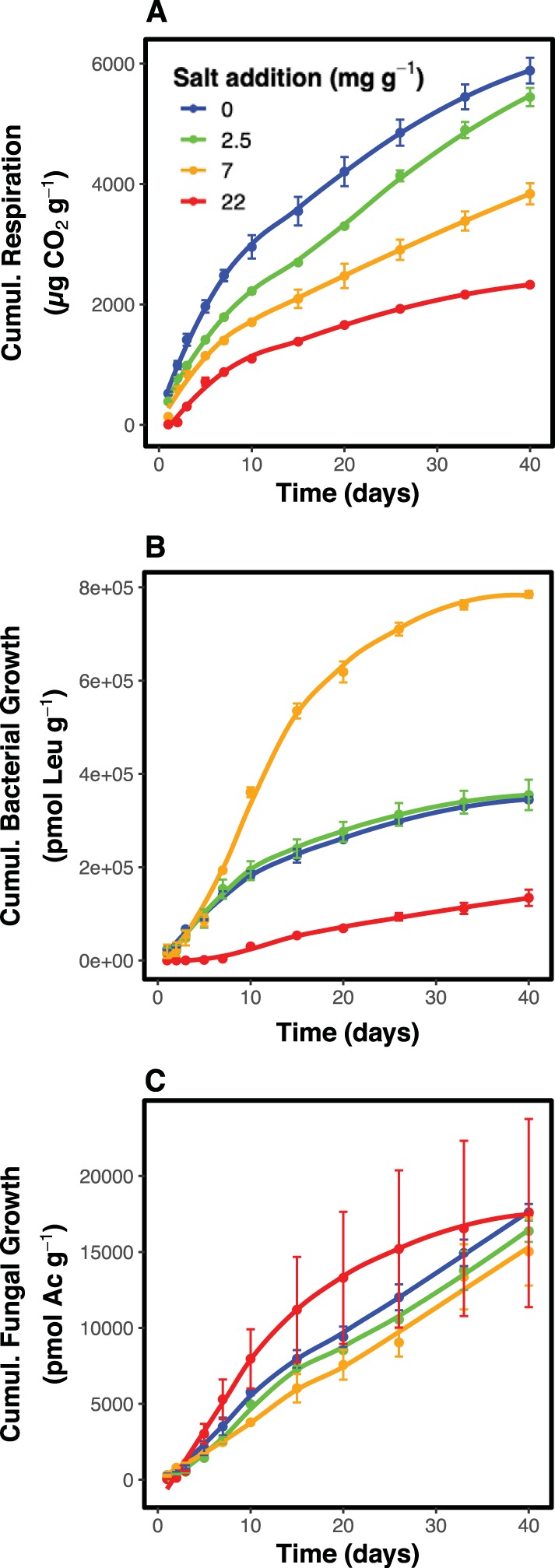
Cumulative respiration (A), bacterial growth (B) and fungal growth (C) rates in microcosms of four different salt treatments (0, 2, 7, and 22 mg NaCl g^−1^) with time after salt addition (in days). Values are means ± 1 standard error.

In the 0 and 2 mg NaCl g^−1^ treatments, respiration was highest on the first day of the experiment and then decreased exponentially over the course of the next 40 days (Fig. 3A). On day 1, respiration was higher in the 0 mg NaCl g^−1^ treatment than in the 2 mg NaCl g^−1^ treatment, but later on, respiration rates converged to similar values in the two treatments. In the 7 mg NaCl g^−1^ and 22 mg NaCl g^−1^ treatments, respiration rates were low initially and reached their highest rates on days 2 and 3, respectively. Afterwards the respiration rate declined exponentially. At the end of the experiment, on day 40, respiration rates were lower in the 22 mg NaCl g^−1^ treatment than in the other treatments. These dynamics translated into values for cumulative respiration over the 40-day study period that decreased with higher salinity from ca. 6,000 μg CO_2_ g^−1^ soil in the 0 mg NaCl g^−1^ treatment to ca. 2,000 μg CO_2_ g^−1^ soil in the 2 mg g^−1^ treatment (Fig. 4A).

Bacterial growth rates in the 0 and 2 mg NaCl g^−1^ treatments peaked at day 2 and then decreased exponentially (Fig. 3B). In the 7 mg NaCl g^−1^ treatment, bacterial growth rates were similar to those in the 0 and 2 mg NaCl g^−1^ treatments on day 1, but then they increased sharply until day 10. This was followed by an exponential decrease, during which bacterial growth rates in the 7 mg NaCl g^−1^ treatment remained higher than in any other treatment throughout most of the experiment. In the 22 mg NaCl g^−1^ treatment, bacterial growth rates were close to 0 in the beginning of the experiment (Fig. 3B). Bacterial growth rates in the 22 mg NaCl g^−1^ treatment then recovered slightly until day 10, but they never reached rates as high as the peak growth rates in the other treatments. On day 40, bacterial growth rates were similar in all treatments. These dynamics translated into cumulative bacterial growth over the 40-day study period in the 7 mg NaCl g^−1^ treatment that was much higher than in any other treatments, with a more than 200% increase over the cumulative growth in the 0 and 2 mg NaCl g^−1^ treatments (Fig. 4B). Cumulative bacterial growth was lowest in the 22 mg NaCl g^−1^ treatment.

Fungal growth rates took longer to reach their maximum rates than either respiration and bacterial growth (Fig. 3C). Fungal growth rates in the 0 and 2 mg NaCl g^−1^ treatments were similar for much of the experiment. A linear increase was seen until day 10, followed by an exponential decrease. On day 0, fungal growth rates in the 7 mg NaCl g^−1^ treatment were similar to those in the 0 mg NaCl g^−1^ treatment, but after that, the rate of increase was low. The maximum fungal growth rate in the 7 mg NaCl g^−1^ treatment was measured on day 15, and it was lower than the maximum fungal growth rate in any other treatment. In the 22 mg NaCl g^−1^ treatment, fungal growth rates were initially close to 0, but then they increased rapidly and exponentially until day 10, to be followed by an exponential decrease. On day 40, fungal growth was highest in the 0 and 2 mg NaCl g^−1^ treatments and lowest in the 22 mg NaCl g^−1^ treatment. These dynamics translated into cumulative fungal growth values over the 40-day study period that were highest in the 22 mg NaCl g^−1^ treatment and lowest in the 7 mg NaCl g^−1^ treatment (Fig. 4C).

## DISCUSSION

### Using soil salinity as a filter for community trait distributions and community composition.

In accordance with our hypothesis, the exposure to an environmental gradient filtered the targeted community trait distributions. Salt exposure induced an increased community salt tolerance in all treatments with added salt (Fig. 1). In addition, the level of salt tolerance increased with the amount of salt that communities were exposed to (Fig. 1). A higher bacterial tolerance to salt had begun to manifest within only a few days after salt exposure, which suggested that microbial communities could quickly align their functional trait distributions in response to changing environmental conditions. Within about a week, the level of salt tolerance stabilized for treatments up to 7 mg NaCl g^−1^ upon reaching a level matched to its new environment (Fig. 1B). It took about 10 days longer in the 22 mg NaCl g^−1^ treatment to reach a constant level of community salt tolerance than in the less saline treatments, thus supporting our hypothesis that the alignment between trait distributions and environment was gradual and would take longer to stabilize for stronger environmental filters. However, it should be noted that in this study communities were supplied with ample resources to colonize during their adjustment to a new environment through the addition of plant material. Since the mechanisms underlying the alignment of the community salt tolerance to the salinity of the environment require both energy and carbon for the synthesis of, e.g., compatible osmolytes (7, 27) or resources required for a new tolerant community to form, it is possible that under stronger resource limitation, the induction of community tolerance to salt would be slower to develop. Despite a relatively minor impact of salinity on respiration and growth in the 2 mg NaCl g^−1^ treatment (Fig. 3 and 4), an increased community salt tolerance was induced also in this treatment. This shift in trait distributions was further accompanied by a shift in bacterial community composition (Fig. 2), suggesting that physiological change of the original community alone was insufficient to explain the trait distribution change and that community turnover played an important role (28).

As the soil used in this experiment had no history of salt exposure, it is likely that the initial species pool contained few or no salt-tolerant species compared to a community that had been exposed to high salinity previously (29). This starting pool of species is likely to have limited the degree of salt tolerance achievable in the community. In addition, in natural systems, dispersal could bring in more salt-adapted species from surrounding localities, promoting the establishment of a more salt-tolerant local community (30). Put differently, it could be expected that our study system was underdispersed. By comparing the levels of salt tolerance acquired within this experiment to those described for natural long-term gradients of salinity (10), inferences can be drawn about whether dispersal had limited the level of trait distributions attainable within our study system. In the 2 and 7 mg NaCl g^−1^ treatments (corresponding to saline and medium saline soil environments), the limitations of the species pool and the lack of dispersal were probably not important limitations for the development of community salt tolerance, since far higher levels could be reached at the 22 mg NaCl g^−1^ treatment (Fig. 1) (a level of salt corresponding to hypersaline soil), demonstrating substantial remaining potential for trait distributions to change further. However, it is possible that at salinities similar to those reached in the 22 mg NaCl g^−1^ treatment, a higher community salt tolerance could have been achieved with a different species pool (10). Along natural salinity gradients, salt community tolerance in soils of similar salinities as indicated by the IC_50_ was found to be about a factor of 1.5 to 2.5 higher, ranging between ca. 1,000 mM to 1,500 mM NaCl in suspension (10), compared to the IC_50_ of 650 mM in the 22 mg NaCl g^−1^ microcosms.

Clear and large differences in bacterial community composition between salt treatments had developed by the end of the experiment, suggesting that shifts in community trait distributions (salt tolerance) were mostly driven by shifts in community composition, with more salt-tolerant species replacing less salt-tolerant ones. The visible arch effect (Fig. 2A), where an underlying linear gradient is presented as a curve in the ordination, is a common phenomenon in ordination methods when species gradually replace each other along environmental gradients (31, 32). In addition, the arch effect grows stronger the larger the dissimilarities are within the studied communities, a phenomenon that emphasized the extreme community dissimilarities that separated the low and high ends of the experimental salinity gradient, stressing that species turnover was an important contributor mechanism by which the trait distribution changes observed could be explained. By using a nonsaturating distance metric (31), the arch effect could be removed (Fig. 2C), and the dominant influence of treatment salinity on bacterial community composition became more strongly apparent. Many of the bacterial OTUs positively linked to increased community salt tolerance belonged to the phylum *Firmicutes*. In addition to including several halophilic and halotolerant strains, *Firmicutes* include many species that form endospores and are thus able to survive in extreme environments, among those many species known to be salt tolerant (33). The ability to form spores would have given *Firmicutes* an advantage to survive the acute effects of salt exposure and grow more abundant after other bacteria had died off.

### Consequences of the environmental filter on microbial functions.

Respiration increased more rapidly after the addition of substrate than did bacterial and fungal growth (Fig. 3), which suggested that added substrate was initially used for respiration rather than biosynthesis. The discrepancy between the onset of respiration and the onset of growth was especially apparent in the treatments receiving ≥7 mg NaCl g^−1^ (Fig. 3). It is possible that as energy costs required for salt adaptation mechanisms increased, less substrate could be allocated to the synthesis of new biomass (7, 27). Despite differences in the fungal and bacterial contribution to growth between salt treatments (see the next section), respiration was not related to changes in the relative importance of fungi and bacteria but rather to salinity directly (Fig. 4). These results suggest a high degree of functional redundancy within microbial communities (28, 30), extending even to the division of fungi and bacteria. However, all measured functions (fungal growth, bacterial growth, and respiration) were severely inhibited the first few days after the exposure to elevated salt concentrations (Fig. 3). With regard to the bacterial community, it was only when the level of salt tolerance (Fig. 1) had been allowed to adjust to the new environment via species sorting (Fig. 2) that bacterial growth started to recover (approximately on day 7; Fig. 1B and 3B). These links between responses in trait distributions, changes in community composition and the resulting functions of the bacterial community showcase that trait-based approaches can make structural information predictive (25, 28–30, 34, 35). Although initially similarly compromised, the fungal community derived from the nonsaline soil sample recovered faster than did bacteria (Fig. 3C), and the recovery of fungal growth rates were closely matched to the overall decomposer functioning, as indicated by respiration (Fig. 3A). These patterns suggested that (i) fungi were faster to shift their community trait distributions to a new environment (see the next section) and that fungal growth was rate-limiting for the decomposer functioning of our study system.

### Consequences of the environmental filter on the relative importance of fungi to bacteria.

We expected that both bacterial and fungal growth would decrease with increasing salinity as a consequence of the inhibiting effect of salinity on growth (6). However, our results showed a more complicated pattern of growth response to salinity that did not confirm our hypothesis (Fig. 3 and 4). Bacterial growth was highest in the 7 mg NaCl g^−1^ treatment and lowest in the 22 mg NaCl g^−1^ treatment and vice versa for fungal growth (Fig. 3 and 4). There was a negative relationship between fungal and bacterial growth, likely reflecting a competitive interaction between bacteria and fungi. During decomposition, fungi and bacteria compete for substrate, and it has been reported that the activity of bacteria has an antagonistic effect on fungi (36, 37). As a result, suppression of bacterial growth in the 22 mg NaCl g^−1^ treatment by salinity could have resulted in a promotion of fungal growth, as fungi were released from competition with bacteria (38). In contrast, in the 7 mg NaCl g^−1^ treatment, fungal growth was likely suppressed by high bacterial growth.

The fact that the fungal community derived from a nonsaline soil could grow in the 22 mg NaCl g^−1^ treatment where the growth of the corresponding bacterial community remained low throughout the experiment (Fig. 3 and 4) corroborates our hypothesis that fungal communities have a higher potential to tolerate increases in salinity than bacterial communities do. This is in accordance with previous results characterizing various microbial processes in an initially nonsaline soil, finding less inhibition of fungal growth compared to bacterial growth in response to salt exposure (6), as well as studies that found an increase in the fungal/bacterial ratio of biomass in saline soils (11, 12). The chitinous cell walls of fungi provide better protection against water loss caused by low soil moisture (39, 40) and could conceivably also contribute to the higher resistance of fungi to low water potential caused by high solute concentrations (41, 42). Increased fungal resistance has also been found in response to high heavy metal exposure (43) and low pH (38) in soils, two disturbances that also result in high extracellular cation concentrations. The interpretation is complicated by the fact that in the 7 mg NaCl g^−1^ soil, bacterial growth clearly dominated (Fig. 3 and 4). Possibly, fast growing opportunistic bacteria took over in that treatment after severe disturbance of the community present in the soil before the addition of salt, and consequently suppressed the initially high fungal growth. In the 0 and 2 mg NaCl g^−1^ treatments, fungal and bacterial growth were both at intermediate levels (Fig. 3 and 4), since salt exposure did not lead to disruption of either group, and consequently did not result in the competitive release of fungi or bacteria.

We combined two different plant litters to equally stimulate both bacterial and fungal growth, as alfalfa promotes bacterial growth, while wheat straw promotes fungal growth (44, 45). In those treatments in which bacteria could establish, bacterial growth increased more rapidly in response to plant material addition than did fungal growth (Fig. 4), which could reflect the different ecological strategies of bacteria and fungi, since bacteria are commonly associated with an r-selected strategy and fungi with a K-selected strategy for resource use (15, 46, 47). Due to higher growth rates on new resources, bacteria would thus have been faster to respond after substrate addition.

### Conclusion.

Our findings suggest that microbial communities can quickly adapt to fluctuating salt concentrations in the soil and that the shifting community salt tolerance is partly driven by changes in community composition. When bacterial salt tolerance had been induced, this was accompanied by the recovery of the microbial functions respiration and growth. We found suggestions for competitive interactions of fungi and bacteria during decomposition of plant litter. Fungal communities derived from the nonsaline soil could resist higher salt concentrations than the corresponding bacterial communities, and the resulting balance between fungal and bacterial decomposers in saline soils was thus a function of both abiotic and biotic factors.

## MATERIALS AND METHODS

### Soil.

Soil samples were collected in May 2015 from a grassland site in Vomb, Southern Sweden (55° 40' 27'' N, 13° 32' 45'' E). Soil samples were collected from the Ah horizon (0 to 15 cm, sandy loam) of a freely draining, grassland soil classified as a Eutric cambisol (48). Multiple samples were collected and combined into a composite soil sample. Roots were removed, and the soil sample was sieved (<2.8 mm). The soil had a water content (gravimetric, 24 h at 105°C) of ca. 28% ± 0.4% dry weight (dw) (mean ± 1 standard error [SE] of three replicates), a water holding capacity (WHC) of 65% ± 2% dw and an organic carbon content (loss on ignition, 600°C for 12 h) of 19.6% ± 0.6% dw. In a 1:5 soil-water mixture, the pH was 6.1 ± 0.02, and the electrical conductivity (EC) was 0.1 ± 0.01 dS m^−1^.

### Experimental design.

Microcosms were set up with 250 g of soil in 1 l plastic containers with an airtight lid and adjusted to four different levels of salinity (the “environmental filter”) by adding different amounts of NaCl (0, 2, 7, and 22 mg NaCl g^−1^ soil) together with 100 μl of water per g soil. These additions resulted in electrical conductivities in 1:5 soil-water extractions including 0.1, 1.1, 2.8, and 6.8 dS m^−1^ (Table 1), which is equivalent to about 1.1, 6.7, 16, and 38 dS m^−1^ in a saturated paste (49), thus including the categories nonsaline, saline (>4 dS m^−1^), medium saline (8 to 16 dS m^−1^), and hypersaline soil (>35 dS m^−1^ in saturated paste) (50). Duplicate microcosms were prepared for each treatment, and microcosms were supplied with 15 mg g^−1^ soil 1:1 ground alfalfa-wheat straw mixture, in order to provide sufficient resources for rapid adjustment to new salinities and to promote the net growth of a microbial community during the course of the experiment (see below) and therefore for the DNA composition at the endpoint to reflect the traits of the formed community. Over the course of the experiment (40 days), microcosms were incubated at 18°C in the dark, corresponding to summer soil temperatures at the site. The water content in the microcosms was monitored and maintained at a constant level, and microcosms were regularly aerated. On days 1, 2, 3, 5, 7, 10, 15, 20, 26, and 40 after the exposure to salt additions (the environmental filter), respiration (CO_2_ production), bacterial growth (leucine incorporation), and fungal growth (acetate incorporation into ergosterol) were measured in subsamples from each microcosm. In addition, bacterial salt tolerance (the targeted community trait distribution) was determined from dose-response relationships of bacterial growth to salinity (see the section below). Bacterial growth measurements and community tolerance measurements on days 2 and 5 were removed from further analysis due to analytical issues due to low leucine incorporation rates. Soil samples for the determination of bacterial community structure were taken at the end of the experiment on day 40.

**TABLE 1:** Salinity of the four different salt addition treatments

Treatment	NaCl addition (mg g^−1^)	EC (dS m^−1^)^*a*^
I	0	0.1 ± 0.02
II	2	1.1 ± 0.04
III	7	2.8 ± 0.07
IV	22	6.8 ± 0.43

a Electrical conductivity (EC) values are given as the mean ± 1 standard error for three technical replicates.

### Microbial functions.

Bacterial growth was measured as the incorporation of ^3^H-labeled leucine into bacterial biomass in bacteria extracted from soil by the methods of E. Bååth (51) and E. Bååth et al. (52). Briefly, soil suspension was created by mixing 1 g of soil with 20 ml of water followed by a centrifugation at 1,000 × *g* for 10 min. A 1.35 ml subsample was taken from the suspension. ^3^H-labeled leucine (2 μl; 37 MBq ml^−1^ and 5.74 TBq mmol^−1^; Perkin Elmer, UK) was added to the suspension together with nonlabeled leucine, resulting in a final concentration of 275 nM Leu. After 1 h of incubation at 18°C in the dark, growth was terminated by the addition of 100% trichloroacetic acid. After a series of washing steps, the amount of incorporated ^3^H-label was measured using liquid scintillation.

Fungal growth was estimated using the incorporation of ^14^C-labeled acetate into ergosterol by the method of E. Bååth (53) with the following modifications. Subsamples of soil (1 g) were mixed with 2 ml of water to create a soil slurry. To the soil slurry, 20 μl of [1-^14^C]acetic acid (sodium salt, 37 MBq ml^−1^, 2.10 GBq mmol^−1^) (Perkin Elmer) was added combined with 30 μl of 16 mM nonlabeled sodium acetate, resulting in a final concentration of ca. 220 μM sodium acetate. Samples were incubated at 18°C in the dark for 4 h, after which growth was terminated using 1 ml of 5% formalin. Ergosterol was then extracted from the samples, separated using high-performance liquid chromatography and a UV detector, and collected in a fraction collector. The radioactivity in the sample was measured using liquid scintillation.

Respiration was determined in subsamples of soil (1 g) that were weighed in 20-ml glass vials, purged with pressurized air, sealed, and incubated for ca. 16 h at 18°C in the dark. Afterwards the CO_2_ concentration in the headspace was analyzed using a gas chromatograph (GC), equipped with a methanizer and a flame ionization detector. The background levels of CO_2_ in pressurized air were subtracted from CO_2_ concentrations measured in the sample headspace.

To establish estimates of bacterial salt tolerance (the targeted community trait) dose-response relationships for bacterial growth and salt were determined. Soil suspensions were created as described above. From each sample, subsamples of 1.35 ml suspension were taken and mixed with 0.15 ml of different NaCl solutions with concentrations of 0, 0.008, 0.02, 0.07, 0.22, 0.67, 2.0, and 6.0 mol/liter of NaCl. These aliquots of bacterial suspension were preincubated with salt treatments for 30 min before bacterial growth was estimated with leucine incorporation, as described above, generating a dose-response relationship and thus estimates of salinity tolerance (see below). Earlier studies have shown that the neither the dilution involved with the homogenization step nor the osmotic shock induced by salt addition require acclimation time before growth rates can be determined (6, 54), and work on other toxicants (55, 56) and both pilot experiments and earlier studies with NaCl (57) showed that 30-min preincubation before leucine incorporation determination was sufficient.

### Soil bacterial community structure.

The composition of the bacterial community was determined through sequencing of the bacterial 16S rRNA gene. DNA was extracted from portions of 250 mg of homogenized ground soil using the MoBio PowerSoil DNA isolation kit (Carlsbad, CA, USA) according to the manufacturer’s recommendations. Extracted DNA was amplified using the 16S rRNA gene primer pair 515-F (5′-GTGCCAGCMGCCGCGGTAA-3′) and 806-R (5′-GGACTACHVGGGTWTCTAAT-3′), which included Illumina adapters and unique barcode sequences for each sample. PCR was performed with GoTaq Hot Start PCR Master Mix (Promega, Madison, WI, USA) in a 25-μl reaction mixture. Thermal cycling consisted of an initial denaturation step at 94°C for 3 min, followed by 35 cycles, with 1 cycle consisting of denaturation at 94°C (45 s), annealing at 50°C (30 s),and extension at 70°C (90 s), and a final extension step at 72°C for 10 min. The amplified DNA was sequenced using an Illumina MiSeq platform (Illumina, San Diego, CA USA).

Sequences were processed and clustered into operational taxonomic units (OTUs) using the UPARSE pipeline (58) by the method of K. S. Ramirez et al. (59). Sequences were quality filtered and clustered *de novo* into OTUs at a 97% similarity level. Taxonomic information was assigned to OTUs using the 16S rRNA Greengenes database as implemented in QIIME 1.9.1 (60). To correct for differences in sequencing depth, samples were rarefied to 10,000 reads. OTUs that were observed fewer than 10 times across all samples were excluded from downstream analyses.

### Data analysis.

To assess the targeted community trait distribution—salt tolerance, bacterial growth estimates measured across the NaCl dilution curves as described in the section above were averaged between samples from replicate microcosms and normalized to the value at the salinity level that led to the maximum growth rate for that sample. This normalization resulted in normalized bacterial growth values ranging between 0 (total inhibition) and 1 (no inhibition). Dose-response relationships were established between the logarithm of NaCl concentration in the suspensions and normalized bacterial growth using a logistic model (57), *Y = c*/[1*+ e^b^*^(^*^x-a^*^)^], where *Y* is the leucine incorporation rate, *x* is the logarithm of the salt concentration in the suspension, *a* is the IC_50_, *c* is the bacterial growth rate without salt inhibition, and *b* is a slope parameter indicating the rate of inhibition. The IC_50_ denotes the salt concentration at which bacterial growth is inhibited by 50% compared to uninhibited growth and provides an index for the targeted community trait distribution, where the IC_50_ level corresponds to salt tolerance (i.e., higher values mean higher tolerance). Estimates of variance of the IC_50_ were obtained from the standard error of the coefficient *a* in the model fit.

Shifts in the bacterial community composition were visualized using principal coordinate analysis (PCoA) based on Bray-Curtis dissimilarities after Hellinger transformation of data. Since a strong arch effect was visible in the ordination, an alternative method was tested using a nonsaturating distance metric, Earth Mover Band Aware Distance (EMBAD), by the method of J. T. Morton et al. (31). The first axes of the PCoAs were then regressed against community salt tolerance. A Mantel test with 1,000 permutations was performed using Spearman’s correlation coefficient to test for a significant correlation between the community composition distance matrices and a Euclidean distance matrix of community salt tolerance. OTUs with an abundance of >0.5% of reads in at least one sample were correlated with community salt tolerance using Spearman’s correlation to identify abundant OTUs positively correlated (ρ > 0.5) with community salt tolerance. EMBAD was calculated using the PyEMD package (61); other statistical analyses of the bacterial community composition were carried out in R 3.5.0 using the *vegan* package (62).

### Data availability.

Sequence data have been deposited at NCBI under BioProject accession no. PRJNA549118.

10.1128/mBio.01607-19.2 FIG S1 Salt tolerance curves for bacterial growth for each sample and time point. Data points represent the mean of two replicate samples for each treatment and salt concentration, while error bars indicate he standard errors of the means. On day 3, due to high variability of measurements, no inhibition curves could be fit to samples from the 2 and 0 mg NaCl g^−1^ treatments. Download FIG S1, DOCX file, 2.4 MB. Copyright © 2019 Rath et al. 2019 Rath et al. This content is distributed under the terms of the Creative Commons Attribution 4.0 International license.

## References

[1] Rengasamy P 2006 World salinization with emphasis on Australia. J Exp Bot 57:1017–1023. doi:10.1093/jxb/erj108. 16510516

[2] Food and Agriculture Organization. 2011 The state of the world’s land and water resources for food and agriculture: managing systems at risk, 1st ed. Earthscan, New York, NY.

[3] Jobbagy EG, Jackson RB 2004 Groundwater use and salinization with grassland afforestation. Global Change Biol 10:1299–1312. doi:10.1111/j.1365-2486.2004.00806.x.

[4] Rath KM, Rousk J 2015 Salt effects on the soil microbial decomposer community and their role in organic carbon cycling: a review. Soil Biol Biochem 81:108–123. doi:10.1016/j.soilbio.2014.11.001.

[5] Setia R, Marschner P, Baldock J, Chittleborough D, Smith P, Smith J 2011 Salinity effects on carbon mineralization in soils of varying texture. Soil Biol Biochem 43:1908–1916. doi:10.1016/j.soilbio.2011.05.013.

[6] Rath KM, Maheshwari A, Bengtson P, Rousk J 2016 Comparative toxicities of salts on microbial processes in soil. Appl Environ Microbiol 82:2012–2020. doi:10.1128/AEM.04052-15. 26801570 PMC4807522

[7] Kakumanu ML, Williams MA 2014 Osmolyte dynamics and microbial communities vary in response to osmotic more than matric water deficit gradients in two soils. Soil Biol Biochem 79:14–24. doi:10.1016/j.soilbio.2014.08.015.

[8] Turk M, Montiel V, Žigon D, Plemenitaš A, Ramos J 2007 Plasma membrane composition of Debaryomyces hansenii adapts to changes in pH and external salinity. Microbiology 153:3586–3592. doi:10.1099/mic.0.2007/009563-0. 17906155

[9] Zhang Y-M, Rock CO 2008 Membrane lipid homeostasis in bacteria. Nat Rev Microbiol 6:222–233. doi:10.1038/nrmicro1839. 18264115

[10] Rath KM, Fierer N, Murphy DV, Rousk J 2019 Linking bacterial community composition to soil salinity along environmental gradients. ISME J 13:836–846. doi:10.1038/s41396-018-0313-8. 30446737 PMC6461869

[11] Kamble PN, Gaikwad VB, Kuchekar SR, Bååth E 2014 Microbial growth, biomass, community structure and nutrient limitation in high pH and salinity soils from Pravaranagar (India). Eur J Soil Biol 65:87–95. doi:10.1016/j.ejsobi.2014.10.005.

[12] Wichern J, Wichern F, Joergensen RG 2006 Impact of salinity on soil microbial communities and the decomposition of maize in acidic soils. Geoderma 137:100–108. doi:10.1016/j.geoderma.2006.08.001.

[13] Sardinha M, Muller T, Schmeisky H, Joergensen RG 2003 Microbial performance in soils along a salinity gradient under acidic conditions. Appl Soil Ecol 23:237–244. doi:10.1016/S0929-1393(03)00027-1.

[14] Pankhurst CE, Yu S, Hawke BG, Harch BD 2001 Capacity of fatty acid profiles and substrate utilization patterns to describe differences in soil microbial communities associated with increased salinity or alkalinity at three locations in South Australia. Biol Fertil Soils 33:204–217. doi:10.1007/s003740000309.

[15] de Boer W, Folman LB, Summerbell RC, Boddy L 2005 Living in a fungal world: impact of fungi on soil bacterial niche development. FEMS Microbiol Rev 29:795–811. doi:10.1016/j.femsre.2004.11.005. 16102603

[16] Six J, Frey SD, Thiet RK, Batten KM 2006 Bacterial and fungal contributions to carbon sequestration in agroecosystems. Soil Sci Soc Am J 70:555–569. doi:10.2136/sssaj2004.0347.

[17] Mouginot C, Kawamura R, Matulich KL, Berlemont R, Allison SD, Amend AS, Martiny AC 2014 Elemental stoichiometry of Fungi and Bacteria strains from grassland leaf litter. Soil Biol Biochem 76:278–285. doi:10.1016/j.soilbio.2014.05.011.

[18] Strickland MS, Rousk J 2010 Considering fungal:bacterial dominance in soils - methods, controls, and ecosystem implications. Soil Biol Biochem 42:1385–1395. doi:10.1016/j.soilbio.2010.05.007.

[19] Schmidt MWI, Torn MS, Abiven S, Dittmar T, Guggenberger G, Janssens IA, Kleber M, Kogel-Knabner I, Lehmann J, Manning DAC, Nannipieri P, Rasse DP, Weiner S, Trumbore SE 2011 Persistence of soil organic matter as an ecosystem property. Nature 478:49–56. doi:10.1038/nature10386. 21979045

[20] Suding KN, Lavorel S, Chapin FS, Cornelissen JHC, Diaz S, Garnier E, Goldberg D, Hooper DU, Jackson ST, Navas ML 2008 Scaling environmental change through the community-level: a trait-based response-and-effect framework for plants. Global Change Biol 14:1125–1140. doi:10.1111/j.1365-2486.2008.01557.x.

[21] Diaz S, Purvis A, Cornelissen JHC, Mace GM, Donoghue MJ, Ewers RM, Jordano P, Pearse WD 2013 Functional traits, the phylogeny of function, and ecosystem service vulnerability. Ecol Evol 3:2958–2975. doi:10.1002/ece3.601. 24101986 PMC3790543

[22] Allison SD 2012 A trait-based approach for modelling microbial litter decomposition. Ecol Lett 15:1058–1070. doi:10.1111/j.1461-0248.2012.01807.x. 22642621

[23] Raes J, Bork P 2008 Molecular eco-systems biology: towards an understanding of community function. Nat Rev Microbiol 6:693–699. doi:10.1038/nrmicro1935. 18587409

[24] Bier RL, Bernhardt ES, Boot CM, Graham EB, Hall EK, Lennon JT, Nemergut DR, Osborne BB, Ruiz-Gonzalez C, Schimel JP, Waldrop MP, Wallenstein MD 2015 Linking microbial community structure and microbial processes: an empirical and conceptual overview. FEMS Microbiol Ecol 91:fiv113. doi:10.1093/femsec/fiv113. 26371074

[25] Webb CT, Hoeting JA, Ames GM, Pyne MI, Poff NL 2010 A structured and dynamic framework to advance traits-based theory and prediction in ecology. Ecol Lett 13:267–283. doi:10.1111/j.1461-0248.2010.01444.x. 20455917

[26] Fierer N, Barberan A, Laughlin DC 2014 Seeing the forest for the genes: using rnetagenomics to infer the aggregated traits of microbial communities. Front Microbiol 5:614. doi:10.3389/fmicb.2014.00614. 25429288 PMC4228856

[27] Oren A 1999 Bioenergetic aspects of halophilism. Microbiol Mol Biol Rev 63:334–348. 10357854 10.1128/mmbr.63.2.334-348.1999 PMC98969

[28] Berga M, Zha YH, Szekely AJ, Langenheder S 2017 Functional and compositional stability of bacterial metacommunities in response to salinity changes. Front Microbiol 8:948. doi:10.3389/fmicb.2017.00948. 28642735 PMC5463035

[29] Vass M, Langenheder S 2017 The legacy of the past: effects of historical processes on microbial metacommunities. Aquat Microb Ecol 79:13–19. doi:10.3354/ame01816.

[30] Shade A, Peter H, Allison SD, Baho DL, Berga M, Burgmann H, Huber DH, Langenheder S, Lennon JT, Martiny JBH, Matulich KL, Schmidt TM, Handelsman J 2012 Fundamentals of microbial community resistance and resilience. Front Microbiol 3:417. doi:10.3389/fmicb.2012.00417. 23267351 PMC3525951

[31] Morton JT, Toran L, Edlund A, Metcalf JL, Lauber C, Knight R 2017 Uncovering the horseshoe effect in microbial analyses. mSystems 2:e00166-16. doi:10.1128/mSystems.00166-16. 28251186 PMC5320001

[32] James FC, McCulloch CE 1990 Multivariate analysis in ecology and systematics: panacea or Pandora’s box. Annu Rev Ecol Syst 21:129–166. doi:10.1146/annurev.ecolsys.21.1.129.

[33] Horikoshi K, Antranikian G, Bull AT, Robb FT, Stetter KO (ed). 2011 Extremophiles handbook. Springer, Tokyo, Japan.

[34] Chase AB, Gomez-Lunar Z, Lopez AE, Li JH, Allison SD, Martiny AC, Martiny J 2018 Emergence of soil bacterial ecotypes along a climate gradient. Environ Microbiol 20:4112–4126. doi:10.1111/1462-2920.14405. 30209883

[35] Vivanco L, Irvine IC, Martiny J 2015 Nonlinear responses in salt marsh functioning to increased nitrogen addition. Ecology 96:936–947. doi:10.1890/13-1983.1. 26230015

[36] Mille-Lindblom C, Fischer H, Tranvik LJ 2006 Antagonism between bacteria and fungi: substrate competition and a possible tradeoff between fungal growth and tolerance towards bacteria. Oikos 113:233–242. doi:10.1111/j.2006.0030-1299.14337.x.

[37] Rousk J, Demoling LA, Bahr A, Baath E 2008 Examining the fungal and bacterial niche overlap using selective inhibitors in soil. FEMS Microbiol Ecol 63:350–358. doi:10.1111/j.1574-6941.2008.00440.x. 18205814

[38] Rousk J, Brookes PC, Bååth E 2010 Investigating the mechanisms for the opposing pH relationships of fungal and bacterial growth in soil. Soil Biol Biochem 42:926–934. doi:10.1016/j.soilbio.2010.02.009.

[39] Manzoni S, Schimel JP, Porporato A 2012 Responses of soil microbial communities to water stress: results from a meta-analysis. Ecology 93:930–938. doi:10.1890/11-0026.1. 22690643

[40] Lennon JT, Aanderud ZT, Lehmkuhl BK, Schoolmaster DR 2012 Mapping the niche space of soil microorganisms using taxonomy and traits. Ecology 93:1867–1879. doi:10.1890/11-1745.1. 22928415

[41] Reischke S, Rousk J, Bååth E 2014 The effects of glucose loading rates on bacterial and fungal growth in soil. Soil Biol Biochem 70:88–95. doi:10.1016/j.soilbio.2013.12.011.

[42] Griffiths BS, Ritz K, Ebblewhite N, Dobson G 1998 Soil microbial community structure: effects of substrate loading rates. Soil Biol Biochem 31:145–153. doi:10.1016/S0038-0717(98)00117-5.

[43] Rajapaksha RMCP, Tobor-Kaplon MA, Bååth E 2004 Metal toxicity affects fungal and bacterial activities in soil differently. Appl Environ Microbiol 70:2966–2973. doi:10.1128/AEM.70.5.2966-2973.2004. 15128558 PMC404458

[44] Rousk J, Bååth E 2007 Fungal and bacterial growth in soil with plant materials of different C/N ratios. FEMS Microbiol Ecol 62:258–267. doi:10.1111/j.1574-6941.2007.00398.x. 17991019

[45] Grosso F, Bååth E, De Nicola F 2016 Bacterial and fungal growth on different plant litter in Mediterranean soils: effects of C/N ratio and soil pH. Appl Soil Ecol 108:1–7. doi:10.1016/j.apsoil.2016.07.020.

[46] Wardle DA, Walker LR, Bardgett RD 2004 Ecosystem properties and forest decline in contrasting long-term chronosequences. Science 305:509–513. doi:10.1126/science.1098778. 15205475

[47] Laliberte E, Kardol P, Didham RK, Teste FP, Turner BL, Wardle DA 2017 Soil fertility shapes belowground food webs across a regional climate gradient. Ecol Lett 20:1273–1284. doi:10.1111/ele.12823. 28853198

[48] IUSS Working Group WRB. 2015 World reference base for soil resources 2014. International soil classification system for naming soils and creating legends for soil maps. Update 2015. World Soil Resources Reports 106. Food and Agriculture Organization of the United Nations, Rome, Italy.

[49] Khorsandi F, Yazdi FA 2011 Estimation of saturated paste extracts’ electrical conductivity from 1:5 soil/water suspension and gypsum. Commun Soil Sci Plant Anal 42:315–321. doi:10.1080/00103624.2011.538885.

[50] Brouwer C, Goffeau A, Heibloem M 1985 Irrigation water management: training manual no. 1 - introduction to irrigation. Food and Agriculture Organization of the United Nations (FAO), Rome, Italy.

[51] Bååth E 1994 Thymidine and leucine incorporation in soil bacteria with different cell size. Microb Ecol 27:267–278. doi:10.1007/BF00182410. 24190340

[52] Bååth E, Pettersson M, Soderberg KH 2001 Adaptation of a rapid and economical microcentrifugation method to measure thymidine and leucine incorporation by soil bacteria. Soil Biol Biochem 33:1571–1574. doi:10.1016/S0038-0717(01)00073-6.

[53] Bååth E 2001 Estimation of fungal growth rates in soil using C-14-acetate incorporation into ergosterol. Soil Biol Biochem 33:2011–2018. doi:10.1016/S0038-0717(01)00137-7.

[54] Rath KM, Maheshwari A, Rousk J 2017 The impact of salinity on the microbial response to drying and rewetting in soil. Soil Biol Biochem 108:17–26. doi:10.1016/j.soilbio.2017.01.018.

[55] Rousk J, Demoling LA, Baath E 2009 Contrasting short-term antibiotic effects on respiration and bacterial growth compromises the validity of the selective respiratory inhibition technique to distinguish fungi and bacteria. Microb Ecol 58:75–85. doi:10.1007/s00248-008-9444-1. 18797957

[56] Rousk J, Ackermann K, Curling SF, Jones DL 2012 Comparative toxicity of nanoparticulate CuO and ZnO to soil bacterial communities. PLoS One 7:e34197. doi:10.1371/journal.pone.0034197. 22479561 PMC3315546

[57] Rousk J, Elyaagubi FK, Jones DL, Godbold DL 2011 Bacterial salt tolerance is unrelated to soil salinity across an arid agroecosystem salinity gradient. Soil Biol Biochem 43:1881–1887. doi:10.1016/j.soilbio.2011.05.007.

[58] Edgar RC 2013 UPARSE: highly accurate OTU sequences from microbial amplicon reads. Nat Methods 10:996–998. doi:10.1038/nmeth.2604. 23955772

[59] Ramirez KS, Leff JW, Barberan A, Bates ST, Betley J, Crowther TW, Kelly EF, Oldfield EE, Shaw EA, Steenbock C, Bradford MA, Wall DH, Fierer N 2014 Biogeographic patterns in below-ground diversity in New York City’s Central Park are similar to those observed globally. Proc Biol Sci 281:20141988. doi:10.1098/rspb.2014.1988. 25274366 PMC4213626

[60] McDonald D, Price MN, Goodrich J, Nawrocki EP, DeSantis TZ, Probst A, Andersen GL, Knight R, Hugenholtz P 2012 An improved Greengenes taxonomy with explicit ranks for ecological and evolutionary analyses of bacteria and archaea. ISME J 6:610–618. doi:10.1038/ismej.2011.139. 22134646 PMC3280142

[61] Pele O, Werman M 2008 A linear time histogram metric for improved SIFT matching, p 495–508. *In* Forsyth D, Torr P, Zisserman A (ed), Computer vision – ECCV 2008. European Conference on Computer Vision. Lecture Notes in Computer Science, vol 5304. Springer, Berlin, Germany.

[62] Oksanen J, Blanchet FG, Friendly M, Kindt R, Legendre P, McGlinn D, Minchin PR, O’Hara RB, Simpson GL, Solymos P, Stevens MHH, Szoecs E, Wagner H 2016 vegan: community ecology package. https://CRAN.R-project.org/package=vegan.

