# Supplementary material for: Linking Microbial Community Structure to Trait Distributions and Functions Using Salinity as an Environmental Filter
Source: mBio. 2019 Jul 23;10(4):e01607-19. doi: 10.1128/mBio.01607-19 (PMC6650560; doi:10.1128/mBio.01607-19)
Supplement: TABLE S1 [file mBio.01607-19-st001.docx]

**Supplemental material**

Table S1:

Table of abundant OTUs (>0.5% of reads) that were positively correlated (Spearman’s rank correlation coefficient ρ >0.5) with bacterial community salt tolerance.

| OTU ID | ρ^1^ | Maximum abundance (% reads)^2^ | Taxonomy |
| --- | --- | --- | --- |
| OTU_1219 | 0.94 | 0.96 | k__Bacteria; p__Firmicutes; c__Bacilli; o__Bacillales; f__; g__; s__ |
| OTU_47 | 0.80 | 1.5 | k__Bacteria; p__Proteobacteria; c__Alphaproteobacteria; o__Rhizobiales; f__Phyllobacteriaceae; g__Aminobacter; s__ |
| OTU_31 | 0.80 | 24 | k__Bacteria; p__Firmicutes; c__Bacilli; o__Bacillales; f__Sporolactobacillaceae; g__; s__ |
| OTU_6238 | 0.80 | 14 | k__Bacteria; p__Proteobacteria; c__Gammaproteobacteria; o__Xanthomonadales; f__Xanthomonadaceae; unclassified; unclassified |
| OTU_229 | 0.79 | 16 | k__Bacteria; p__Firmicutes; c__Bacilli; o__Bacillales; f__Bacillaceae; unclassified; unclassified |
| OTU_342 | 0.79 | 10 | k__Bacteria; p__Actinobacteria; c__Actinobacteria; o__Actinomycetales; f__Brevibacteriaceae; g__Brevibacterium; s__aureum |
| OTU_1140 | 0.79 | 0.95 | k__Bacteria; p__Firmicutes; c__Bacilli; o__Bacillales; f__Paenibacillaceae; unclassified; unclassified |
| OTU_404 | 0.76 | 13 | k__Bacteria; p__Firmicutes; c__Bacilli; o__Bacillales; f__Bacillaceae; g__Gracilibacillus; s__dipsosauri |
| OTU_298 | 0.76 | 12 | k__Bacteria; p__Firmicutes; c__Bacilli; o__Bacillales; f__Bacillaceae; g__Pontibacillus; s__ |
| OTU_893 | 0.76 | 2.3 | k__Bacteria; p__Firmicutes; c__Bacilli; o__Bacillales; unclassified; unclassified; unclassified |
| OTU_1650 | 0.76 | 1.3 | k__Bacteria; p__Actinobacteria; c__Actinobacteria; o__Actinomycetales; f__Dermabacteraceae; g__Brachybacterium; s__ |
| OTU_1837 | 0.76 | 0.69 | k__Bacteria; p__Firmicutes; c__Bacilli; o__Bacillales; unclassified; unclassified; unclassified |
| OTU_545 | 0.73 | 4.5 | k__Bacteria; p__Firmicutes; c__Bacilli; o__Bacillales; unclassified; unclassified; unclassified |
| OTU_293 | 0.66 | 10 | k__Bacteria; p__Bacteroidetes; c__[Saprospirae]; o__[Saprospirales]; f__Chitinophagaceae; g__; s__ |
| OTU_300 | 0.60 | 1.5 | k__Bacteria; p__Verrucomicrobia; c__[Spartobacteria]; o__[Chthoniobacterales]; f__[Chthoniobacteraceae]; g__DA101; s__ |
| OTU_7650 | 0.59 | 6.1 | k__Bacteria; p__Proteobacteria; c__Gammaproteobacteria; o__Xanthomonadales; f__Xanthomonadaceae; g__Rhodanobacter; s__lindaniclasticus |
| OTU_3354 | 0.52 | 2.9 | k__Bacteria; p__Proteobacteria; c__Alphaproteobacteria; o__Rhizobiales; unclassified; unclassified; unclassified |

^1^ Spearman’s rank correlation coefficient

^2^ Maximum proportion of reads in one sample.

^3^ Taxonomy assigned to the OTU sequence: k= kingdom, p= phylum, c=class, o=order, f= family, g=genus, s=species
