# Supplementary material for: Linking Microbial Community Structure to Trait Distributions and Functions Using Salinity as an Environmental Filter
Source: mBio. 2019 Jul 23;10(4):e01607-19. doi: 10.1128/mBio.01607-19 (PMC6650560; doi:10.1128/mBio.01607-19)
Supplement: FIG S1 [file mBio.01607-19-sf001.docx]

Fig S1: Salt tolerance curves for bacterial growth for each sample and time point. Data points represent the mean of two replicate samples for each treatment and salt concentration, while error bars indicate he standard error of the mean. On day 3, due to high variability of measurements no inhibition curves could be fit to samples from the 2 and 0 mg NaCl g^-1^ treatments.
